# Use of profession-role exchange in an interprofessional student team-based community health service-learning experience

**DOI:** 10.1186/s12909-020-02127-z

**Published:** 2020-07-02

**Authors:** Jun Wang, Jie Guo, Yubin Wang, Dan Yan, Juan Liu, Yinghong Zhang, Xianmin Hu

**Affiliations:** College of Medicine, Wuhan University of Science and Technology, Wuhan, China

**Keywords:** Interprofessional education, Profession-role exchange, Role-play, Community service learning, Integration

## Abstract

**Background:**

During interprofessional clinical practice, compared to understanding of one’s own professional role and function, it might be more difficult to clarify the roles and contributions of the other health-care team members because of the inter-professional barrier. In order to provide students the opportunity for real experience with other professions in team environments and enhance their perceptions of other professions’ roles, this study developed a comprehensive and multi-dimension extracurricular interprofessional education (IPE) model through designing and integrating a profession-role exchange component, that was medical students as pharmacists or nurses, pharmacy students as physicians or nurses, and nursing students as physicians or pharmacists in the interprofessional health-care student team, into the service learning experience in a real community setting.

**Methods:**

In this pre/post-intervention study, the effect of integrated profession-role exchange experiences on the students’ attitudes towards interprofessional collaboration and their role clarification was evaluated among 60 student volunteers (20 medical students, 20 pharmacy students and 20 nursing students). All involved students were divided into the profession-role exchange intervention group and the control group. Subjects in the control group did not participate the profession-role exchange experiences, the other IPE procedures were the same for both groups. Three survey instruments for attitudes toward interprofessional clinical collaboration were respectively used to measure the students’ attitudes toward physician-pharmacist, physician-nurse and nurse-pharmacist collaborations. “Roles and responsibilities” subscale of Readiness for Interprofessional Learning Scale was used to evaluate the overall role clarification during IPE.

**Results:**

Compared to the control IPE activity, the addition of profession-role exchange component resulted in the significant increase in students’ positive attitudes towards interprofessional collaboration, and the enhancement of students’ role awareness.

**Conclusions:**

The profession-role exchange might be more effective and better initiate students to the practice of interprofessional collaboration, and could be used as an effective IPE tool for improving the role awareness of health-care students.

## Background

Along with the rapid advancement of modern health-care practices, an interprofessional care model has been well-accepted to be important for supporting a comprehensive patient-centred service provision through blending complementary knowledge and skills from multiple health professions, such as medicine, nursing and pharmacy [1–4]. Through working together in team to care for patients, this interprofessional care model makes possible the best use of resources, improves outcomes, and is able to meet increasingly complex health needs of patients [4]. As an essential element of interprofessional practice and collaboration, clear perceptions of interprofessional team members regarding their professional roles are crucial for the effectiveness of interprofessional care [2, 3]. To successfully participate in a well-functioning interprofessional team, health-care professionals first need to understand the roles of their own and other team members.

The role is considered to represent “a set of expectations in the sense that it is what one *should* do” [2]. The role perceptions of health-care professionals would translated into actual actions in clinical practice, thus are essential for improving patient care and achieving interprofessional team success [2, 3, 5, 6]. Currently, health-care educational models in most regions of the world, for example, in China, focus on uni-professional training [7]. Therefore, health-care professionals have few opportunities to understand the professional characteristics and practices of other professionals during learning and patient care. Therefore, compared to understanding of one’s own professional role and function, it might be more difficult to clarify the roles and contributions of the other health-care team members. *Knowledge of professional role of others* has been mentioned as a key competency that is required for successful interprofessional practice and collaboration [8]. In order to provide a comprehensive and optimal team-based health care, each team member should revolve around specific role expectations and meet perceived professional responsibilities. Team members as a group must have an understanding of the competencies and skills which each team member could contribute, and can determine who is the best suitable one to implement the given clinical intervention that is required in a given situation [2]. Nevertheless, a lack of awareness, definition and recognition of one another’s role was still believed as a main conceptual barrier to interprofessional collaboration as perceived by the involved actors [4, 9, 10], which need to be overcome in the future.

Interprofessional education (IPE), whereby students from different health-care professions learn and practise together, has shown to have a positive impact on team work in the students’ future health-care practice, contribute to train effectual health-care teams and optimize future holistic patient care [10, 11]. In order to prepare the students to interprofessional team practice, health-care education programs should provide adequate learning opportunities that enable students to interact with potential professional team members, gain insight into the roles, professional cultures, and contributions of collaborating team members [2, 11]. Among the varied educational initiatives for teaching interdisciplinary teamwork, community service learning has attracted great interest from health-care educators [7, 12–15]. The service/learner model, a team approach to experiential education, has been suggested as a potential teaching method for IPE [16]. This model challenges the learners to effectively work together to address real clinical problems or patient education in a clinical setting, thus provides a means of linking classroom knowledge with the practical experience [17]. Community service learning is a common representation format for the service/learner model [7, 12–15]. In our previous study [7], we developed a new IPE opportunity through integrating cooperative learning into an interprofessional student team-based community service event. Cooperative learning is an instructional tool to organize students into small groups, in which students work together to help one another learn academic content [7]. In the IPE program previously conducted by our team [7], cooperative learning project was implemented before the community service event, in order to lay the foundation for good communication and shared understanding or thinking among team members. Results showed a significant increase in positive attitudes among the involved students towards interprofessional collaboration after participation in this IPE activity. The use of cooperative learning interacting with colleagues from other professions was beneficial to expand students’ exposure to the role of other health-care professionals [7]. However, this previous IPE opportunity only involved medical and pharmacy students. Importantly, it is generally accepted that role clarification in interprofessional collaboration requires real experience with other professions in team environments [18]. In an effort to improve our existing IPE activity, we further speculated, if some experiential interventions were vividly integrated into the service learning experience in a real community setting, this IPE activity might be more effective and better initiate students to the practice of interprofessional collaboration.

Therefore, we proposed a conceptual framework for profession-role exchange design as a module to provide additional support for IPE. In order to explore a promising approach for enhancing students’ perceptions of the role of other professions and develop a comprehensive and multi-dimension extracurricular IPE model, this study designed and integrated a profession-role exchange component, that was medical students as pharmacists or nurses, pharmacy students as physicians or nurses, and nursing students as physicians or pharmacists, in the interprofessional student team-based community service experience. In this protocol, the students were encouraged to experience interprofessional team working in community settings as a different role from their own professional background. Then, the effect of integrated profession-role exchange experiences on the students’ attitudes towards interprofessional collaboration and their role clarification was evaluated.

## Methods

### Study description

**Fig. 1:**
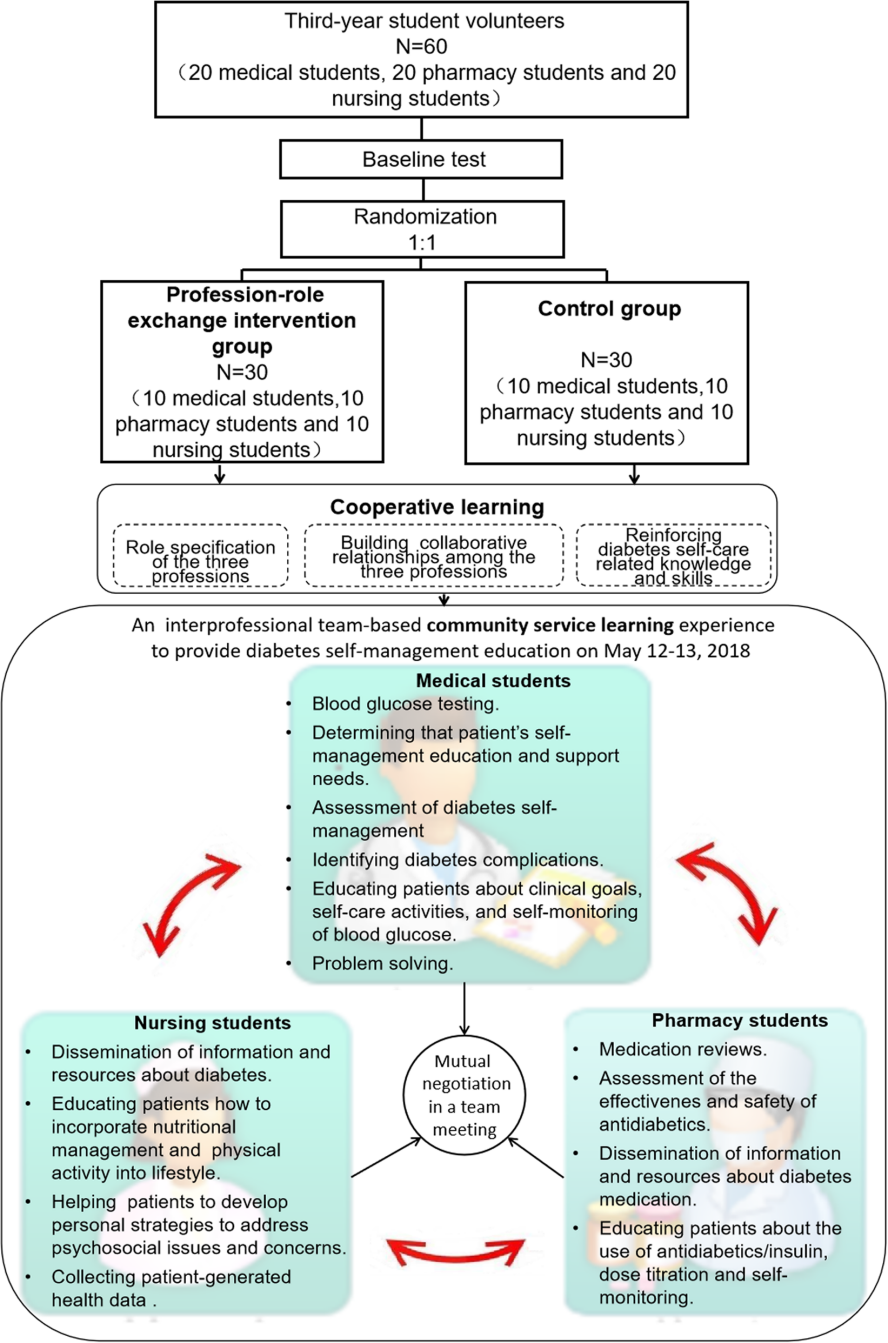
Study flowchart. Subjects in the control group did not participate the profession-role exchange experiences (shown as red arrows), the other IPE procedures were the same for both groups

A total of 60 third-year students (20 medical students, 20 pharmacy students and 20 nursing students; 19 males and 41 females) volunteered to participate in the IPE activity on May 12–13, 2018. They had learned basic disease/drug knowledge and community service skills, and were recruited via the Internet. Faculty organised an interprofessional student team-based local community diabetes self-management education. The involved students were randomly divided into the profession-role exchange intervention group and the control group. Each group was composed of 10 medical students, 10 pharmacy students and 10 nursing students. Two weeks prior to the IPE activity, all the student volunteers were informed about the aim and step of this IPE activity, organized into interprofessional teams during an initial meeting convened by faculty committee. The CONSORT guidelines were followed and the study flowchart is presented in Fig. 1.

#### **Step 1** cooperative learning

Previously, we used cooperative learning based on six-stage model of group investigation to prepare for the subsequent community service learning [7]. The students carried out the cooperative learning in the organized interprofessional teams. Students were arranged to learn and discuss the knowledge and skills, which were required to deliver interprofessional care in the subsequent community service experience, in collaborative small-group learning situations. Moreover, students from different professions were encouraged to offer their own professional opinions about diabetes control, in order to let students from other professions understand their own professional roles and responsibilities. Meanwhile, positive collaborative relationships among the three professions could be built through the reciprocal discussion among team members. On the day before the community service IPE activity, faculty committee convened a formal meeting, in which the students were required to report on their group’s cooperative learning work, present the group community diabetes self-management education project, outline and negotiate all team members’ potential professional contributions.

#### **Step 2** community service learning

Considering the prevalence of increased incidence of type 2 diabetes among the public, the hierarchical diagnosis and treatment system reforms for improving the community health service system have been initiated in response to the challenges of chronic diseases represented by diabetes in China [19]. Accordingly, Chinese health profession students have more opportunities to access to a wide variety of patients with a range of acute to chronic diabetic conditions, and learn professional diabetes-related knowledge and skills in community-based settings. After cooperative learning, the students participated in a community service experience aiming to increase diabetes self-management education. The interprofessional student teams conducted household visits for the community residents suffering from diabetes, the list of whom was given by the community hospital, to educate them about diabetes self-management and address their health-care needs. Each interprofessional student team included 2 medical students, 2 pharmacy students and 2 nursing students. Each group (profession-role exchange intervention group or the control group) included 5 teams to visit the household. The responsibilities of health-care students from different professions were proposed and assigned based on the clinical practice and the Standards for Diabetes Self-Management Education and Support [20](Fig. 1).

### **Intervention** profession-role exchange

On the days the local community diabetes self-management education IPE activity was conducted, in the intervention group, the profession-role exchange experiences were used to further enhance the role awareness of health-care students. As a kind of role-playing education game, profession-role exchange is an innovative simulation- based experiential learning method, in which health-care students from different professions play one another’s role in an environment similar to the clinical environment. This new role experience may help students to develop awareness and valuing of the future collaborating team members’ potential contributions in providing effective patient care, as well as objectively view their own role from the perspective of partners. During the community diabetes self-management education IPE activity (Step 2), students in the intervention group were required to perform the responsibilities of the students from other professions. That is, one medical student in a team should perform the responsibilities of the pharmacy students in their team; the other medical student should play the role of the partner nursing students in their team. And one pharmacy student in this team should act as a student physician; the other pharmacy student should adopt characters or roles of the nursing students in their team, and so on. The role-choices of students were based on their own will and the negotiation among the team members. As students began the role-exchange experiences, they were allowed to consult with their peers, but could not let other students work instead of themselves.

Subjects in the control group did not participate the profession-role exchange experiences, the other IPE procedures (Step 1 and 2) were the same for both groups. The above IPE experience was governed by a volunteer faculty committee, and under the supervision of three qualified practitioners.

### Measures

#### Physician-pharmacist collaboration survey instrument

As common-used validated instrument for measuring interprofessional collaboration between physicians and pharmacists [7, 21], Scale of Attitudes Toward Physician-Pharmacist Collaboration (SATP^2^C) was applied to measure the perceptions of physician-pharmacist collaboration among the involved medical and pharmacy students. There are 16 Likert-type items on a 4-point scale (4:strongly agree; 3:agree; 2: disagree or 1:strongly disagree) in SATP^2^C. Higher scores mean more positive attitudes toward physician-pharmacist collaborative relationships.

#### Physician-nurse collaboration survey instrument

The medical and nursing students in both intervention and control groups were required to complete the Jefferson Scale of Attitudes Towards Physician-Nurse Collaboration (JSAPNC) [22], which included 15 items on a 4-point scale (1:strongly disagree-4:strongly agree). A higher total score indicates a more positive attitude toward physician–nurse collaboration.

#### Nurse-pharmacist collaboration survey instrument

The attitudes toward nurse–pharmacist collaboration among the nursing and pharmacy students were analyzed using a previously self-developed 25-item scale [23], the overall score of which ranges from 25 to 100 theoretically. Higher scores reflect more positive attitudes toward nurse-pharmacist collaboration.

*“Roles and responsibilities” subscale of Readiness for Interprofessional Learning Scale (RIPLS)*

All the participant students were asked for responses to “Roles and responsibilities” subscale of RIPLS, which was firstly developed by Parsell & Bligh [24] to assess the readiness of health-care students for multi-professional learning. There are three underlying factors in RIPLS, which were respectively named as “Team-working and collaboration”, “Professional-identity” and “Roles and responsibilities” [25]. Among them, “Roles and responsibilities” subscale of RIPLS has been widely used to specially evaluate the overall role clarification during IPE [18, 26]. This subscale includes 3 items rated on a 5-point scale, ranging from (1: strongly Disagree - 5: strongly agree). Higher total scores indicate greater role clarification.

The bilingual version of the above survey instruments in Chinese and English was applied in this study to ensure the accurate comprehension of respondents.

### Data collection

At the initial meeting before beginning the IPE intervention, students received a letter of information and the printed questionnaires. Only participants completing the questionnaires were included in the subsequent study. Participants received the same questionnaires immediately following the community service-learning experience. All 60 students completed the pre-study questionnaires, but a medical student in the profession-role exchange intervention group and a nursing student in control group did not complete the post-study questionnaires thus were excluded from analyses.

### Data analysis

**Fig. 2:**
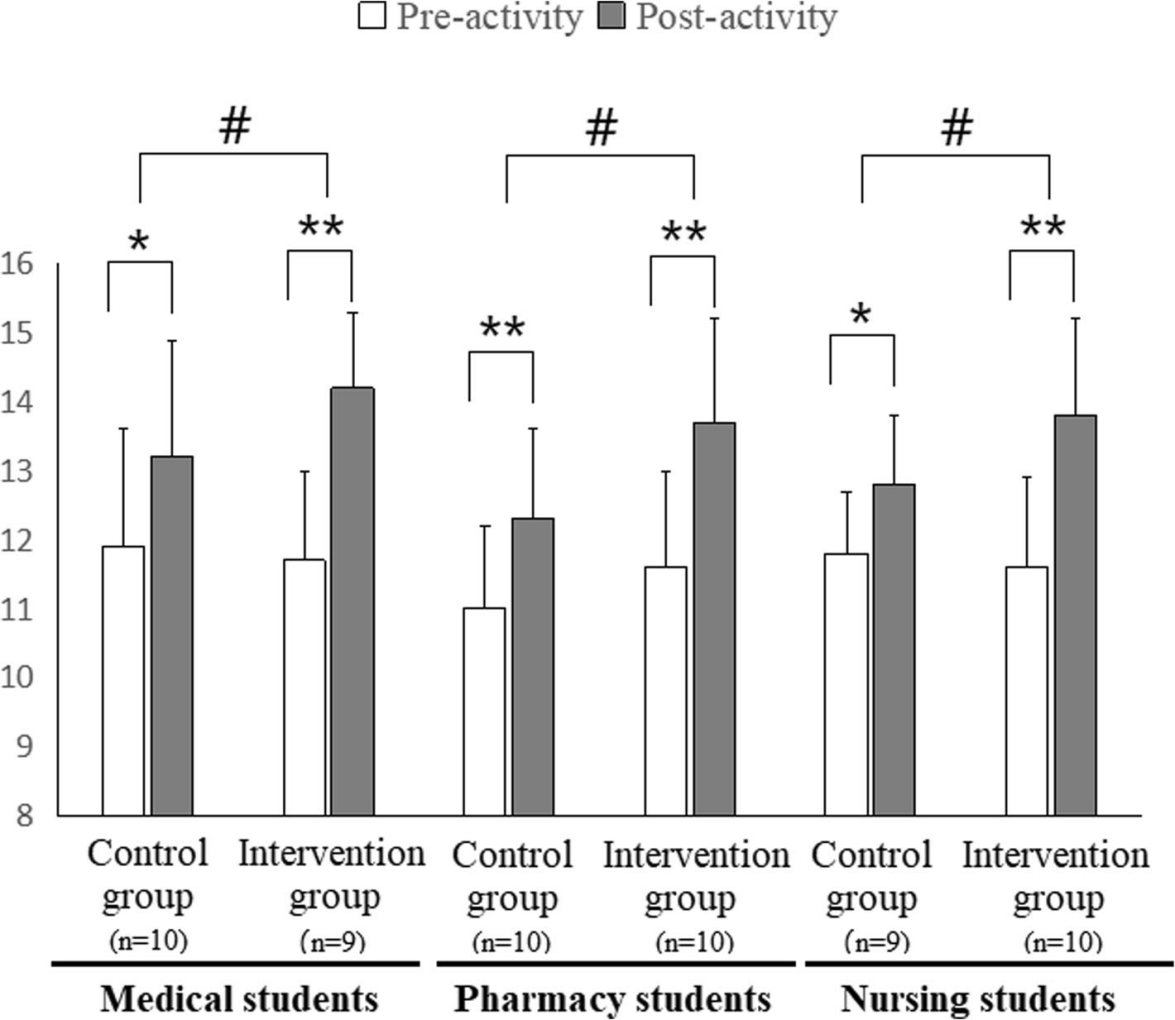
The effect of profession-role exchange intervention on the students’ scores in “Roles and responsibilities” subscale of RIPLS (Mean ± SD). *p < 0.05; **p < 0.01. Pre- and post-activity surveys were analysed using the Wilcoxon matched-pairs signed rank test. # *p* < 0.05. Repeated-measures ANOVA using pre- and post-intervention scores as the within-subject factor and intervention (yes or no) as the between-subject factor was conducted to investigate the differences between the outcomes of the profession-role exchange intervention group and the control group

Data were coded and entered into SPSS22.0. Results were expressed as mean ± standard deviation (SD). Previous studies have identified underlying factors of SATP^2^C [21], JSAPNC [22], Nurse-pharmacist collaboration survey instrument [23], and RIPLS [24] (Tables 1, 2 and 3, Fig. 2). In order to assess the levels of attitudes toward interprofessional collaboration or role clarification and their distribution among different factors, total scores from survey instruments as well as the scores for each extracted factor were recorded. The Wilcoxon matched-pairs signed rank test was used to compare the differences between the results from pre- and post-study surveys. Repeated-measures ANOVA using pre- and post-intervention scores as the within-subject factor and intervention (yes or no) as the between-subject factor was conducted to investigate the differences between the outcomes of the profession-role exchange intervention group and the control group. Differences were considered to be statistically significant when *p*-value < 0.05.

**Table 1:** The effect of profession-role exchange intervention on the students’ attitudes towards physician-pharmacist collaboration measured with SATP^2^C (Mean ± SD)

Factors	Score range (Min.-Max.)	Medical students				Pharmacy students			
		Control group (*n* = 10)		Intervention group (*n* = 9)		Control group (*n* = 10)		Intervention group (*n* = 10)	
		Pre-activity	Post-activity	Pre-activity	Post-activity	Pre-activity	Post-activity	Pre-activity	Post-activity
Responsibility and accountability	9–36	27.8 ± 3.8	31.4 ± 2.6**	28.0 ± 2.9	33.4 ± 2.2**^#^	29.8 ± 2.6	32.6 ± 2.5**	29.2 ± 2.7	33.9 ± 2.3**^#^
Shared authority	5–20	14.9 ± 1.7	18.0 ± 1.5**	15.2 ± 1.7	19.0 ± 1.0**^#^	16.0 ± 1.7	18.3 ± 1.7**	15.6 ± 1.4	18.9 ± 1.3**^#^
Interdisciplinary education	2–8	6.4 ± 1.1	8.4 ± 0.8**	6.7 ± 1.1	9.1 ± 0.8**	6.8 ± 1.1	8.7 ± 1.2**	7.3 ± 1.4	9.3 ± 0.8**
Total scores	16–64	49.1 ± 5.0	57.8 ± 3.4**	49.9 ± 4.3	61.6 ± 2.88**^#^	52.6 ± 3.3	59.6 ± 2.7**	52.1 ± 3.0	62.1 ± 3.7**^#^

*p < 0.05; **p < 0.01. Pre- and post-activity surveys were analysed using the Wilcoxon matched-pairs signed rank test

^#^*p* < 0.05. Repeated-measures ANOVA using pre- and post-intervention scores as the within-subject factor and intervention (yes or no) as the between-subject factor was conducted to investigate the differences between the outcomes of the profession-role exchange intervention group and the control group

**Table 2:** The effect of profession-role exchange intervention on the students’ attitudes towards physician-nurse collaboration measured with JSAPNC (Mean ± SD)

Factors	Score range (Min.-Max.)	Medical students				Nursing students			
		Control group (*n* = 10)		Intervention group (*n* = 9)		Control group (*n* = 9)		Intervention group (*n* = 10)	
		Pre-activity	Post-activity	Pre-activity	Post-activity	Pre-activity	Post-activity	Pre-activity	Post-activity
Shared education and team work	7–28	21.2 ± 1.5	24.7 ± 1.7**	20.8 ± 1.9	24.9 ± 1.9**	21.1 ± 2.0	24.6 ± 1.7**	20.8 ± 1.6	25.2 ± 2.3**
Caring vs. curing	3–12	8.9 ± 1.1	10.6 ± 1.1**	8.9 ± 0.9	11.4 ± 1.3**	9.0 ± 1.4	11.1 ± 1.2**	8.8 ± 1.5	10.7 ± 1.1**
Nurse’s autonomy	3–12	6.0 ± 2.4	7.2 ± 3.0	5.7 ± 1.9	9.3 ± 2.4**^#^	6.4 ± 1.7	8.0 ± 1.7*	6.1 ± 2.0	9.3 ± 2.3**^#^
Physician’s dominance	2–8	3.6 ± 1.5	4.1 ± 2.0	3.9 ± 1.5	6.0 ± 1.9**^#^	4.6 ± 2.1	4.0 ± 1.1	4.7 ± 2.3	7.2 ± 1.3**^#^
Total scores	15–60	39.7 ± 4.0	46.6 ± 4.8**	39.3 ± 3.1	51.7 ± 4.4**^#^	41.1 ± 2.2	47.7 ± 1.9**	40.4 ± 3.0	52.4 ± 3.4**^#^

*p < 0.05; **p < 0.01. Pre- and post-activity surveys were analysed using the Wilcoxon matched-pairs signed rank test

^#^*p* < 0.05. Repeated-measures ANOVA using pre- and post-intervention scores as the within-subject factor and intervention (yes or no) as the between-subject factor was conducted to investigate the differences between the outcomes of the profession-role exchange intervention group and the control group

**Table 3:** The effect of profession-role exchange intervention on the students’ attitudes towards nurse–pharmacist collaboration measured with the nurse-pharmacist collaboration survey instrument (Mean ± SD)

Factors	Score range (Min.-Max.)	Pharmacy students				Nursing students			
		Control group (*n* = 10)		Intervention group (*n* = 10)		Control group (*n* = 9)		Intervention group (*n* = 10)	
		Pre-activity	Post-activity	Pre-activity	Post-activity	Pre-activity	Post-activity	Pre-activity	Post-activity
Interprofessional team- based practice	11–44	30.6 ± 3.8	35.4 ± 5.1**	30.7 ± 4.9	37.3 ± 5.0**	29.4 ± 5.0	35.2 ± 4.8**	29.5 ± 5.1	37.5 ± 2.8**
Roles/responsibilities for collaborative practice	7–28	17.8 ± 6.1	21.2 ± 6.3	17.3 ± 6.4	25.0 ± 1.5**^#^	13.4 ± 5.7	18.4 ± 6.0*	16.0 ± 6.2	24.7 ± 2.5**^#^
Relationship between nurses and pharmacists	4–16	10.4 ± 3.2	12.7 ± 3.0*	11.2 ± 2.0	13.9 ± 1.4**	8.9 ± 2.7	12.2 ± 1.6**	9.6 ± 2.2	12.8 ± 1.7**
Nurses’ experience of pharmacist’s role in drug treatment	3–12	10.4 ± 1.3	11.7 ± 0.7**	10.4 ± 1.6	11.6 ± 0.7*	9.4 ± 1.1	10.3 ± 1.5	8.5 ± 2.2	11.0 ± 1.3**^#^
Total scores	25–100	69.2 ± 9.8	81.0 ± 12.6*	69.6 ± 7.5	87.8 ± 7.1**^#^	61.2 ± 11.2	76.2 ± 9.9**	63.6 ± 6.3	86.0 ± 3.6**^#^

*p < 0.05; **p < 0.01. Pre- and post-activity surveys were analysed using the Wilcoxon matched-pairs signed rank test

^#^*p* < 0.05. Repeated-measures ANOVA using pre- and post-intervention scores as the within-subject factor and intervention (yes or no) as the between-subject factor was conducted to investigate the differences between the outcomes of the profession-role exchange intervention group and the control group

## Results

Because all the participants were the third-year students, their ages were all around 21. Among 29 control students who completed both pre-study and post-study questionnaires, 9 were males and 20 were females. The profession-role exchange intervention group had the same gender distribution as the control group. Collectively, the gender, age, specialty, or grade distribution of participants was approximately equal between the two groups.

### Medical and pharmacy students’ attitudes toward physician-pharmacist collaboration

All the medical and pharmacy participants in the IPE activity, except a medical student, completed both the pre- and post-activity surveys on attitudes toward physician-pharmacist collaboration using SATP^2^C. As shown in Table 1, after the IPE activity, significant enhancements in SATP^2^C scores were found in both control and intervention groups (*p* < 0.01). Importantly, when compared with the control group, the addition of profession-role exchange in the intervention group markedly further increased the total SATP^2^C scores (*p* < 0.05). In the control group, the total SATP^2^C scores were increased by 17.7 and 13.3% for medical and pharmacy students, respectively; in the intervention group, the total scores were respectively increased by 23.4 and 19.2% for medical and pharmacy participants.

Three underlying constructs, namely “Responsibility and accountability”, “Shared authority” and “Interdisciplinary education”, had been emerged from the factor analysis of SATP^2^C [21]. In line with the changes in the total scores, for the medical and pharmacy students, participating in the profession-role exchange was associated with the increased scores in “Responsibility and accountability” and “Shared authority” subscales on the SATP^2^C (*p* < 0.05, vs. control group).

### Medical and nursing students’ attitudes toward physician-nurse collaboration

As shown in Table 2, significant statistical differences (*p* < 0.01) in the total JSAPNC scores were found between pre-activity and post-activity in both control and intervention groups. And the increases of the total score in the medical and nursing students who had experienced profession-role exchange (31.6 and 29.7%, respectively) was more markedly than those of the control students (17.4 and 16.1%, respectively).

Among the four extracted factors of JSAPNC, it was interesting to found that, after the intervention of profession-role exchange, medical and nursing students displayed significantly more positive attitudes toward “Nurse’s autonomy” and “Physician’s dominance” (*p* < 0.05), which are factors reflecting students’ perceptions of professional role. A higher factor score on the “Nurses’ autonomy” dimension suggested a more positive view of nurses’ importance in decisions about patient care [27]. As for two reversely-scored items identified as “Physician’s dominance” questions, more students showed a rejection of physicians’ totally dominant role in patient care after profession-role exchange intervention (*p* < 0.05). These data suggested that the profession-role exchange experiences reversed the common role-stereotype of nurses as “collaborators” and physicians as “team leaders”.

### Nursing and pharmacy students’ attitudes toward nurse–pharmacist collaboration

As shown in Table 3, despite the fact that the control IPE activity increased the total attitude scores toward nurse-pharmacist collaboration among nursing and pharmacy students by 17.1 and 24.5%, respectively (*p* < 0.05; *p* < 0.01, vs. pre-activity), the integration of profession-role exchange experience into the IPE activity achieved further improvement (*p* < 0.05, vs. control group). Importantly, the nursing and pharmacy students’ attitudes toward “Roles/responsibilities for collaborative practice” were significantly enhanced by the profession-role exchange experiences (44.5% in pharmacy students and 54.4% in nursing students of the intervention group vs. 19.1% in pharmacy students and 37.3% in nursing students of control group). And the nursing students’ scores in the “Nurses’ experience of pharmacist’s role in drug treatment” sub-scale were obviously decreased by 29.4% after the profession-role exchange intervention, when compared with those of control nursing students (*p* < 0.05).

### Students’ scores in “Roles and responsibilities” subscale of RIPLS

As shown in Fig. 2, in the control group, the medical, pharmacy and nursing students’ scores in “Roles and responsibilities” subscale of RIPLS were respectively enhanced by 10.9, 11.8 and 8.5% after the control IPE activity (*p* < 0.05; *p* < 0.01). In the profession-role exchange intervention group, the scores of medical, pharmacy and nursing students were enhanced by 21.4, 18.1 and 19.0%, respectively (*p* < 0.05; *p* < 0.01). The between-group comparison showed that participating in the profession-role exchange further increased the scores in the role understanding subscale of RIPLS among students from any of the three health-care professions (*p* < 0.05).

## Discussion

Role-play, as an integral part of simulation-based education method in experiential learning, has been demonstrated to allow for the multi-level brain processing of experiences, thus be effective in constructing the cognitive and emotional learning of health-care students [28, 29]. However, previous educational studies were usually associated with students in single roles during simulation rather than assuming multiple roles [28], which might hinder the students from a comprehensive learning experience in the simulation modality. Therefore, several authors advocated the use of a special kind of role playing named as “role reversal” [30],“dual role-play” [31], or “peer role play” [32] as a teaching strategy whereby health-care students assume multiple roles in role-play simulation. No matter what name it was given, this kind of role-play simulation intervention highlighted the importance of perspective taking in the experiential learning. Nevertheless, all these previous studies [30–32] focused on the switching of roles to experience between health-care professional and patient perspectives. In the present study, we firstly used the role exchange between different professions in the interprofessional student team.

Profession-role exchange in this study offered the health-care students some insight into the experience of being the other professional members in interprofessional clinical team. Due to the multiple roles (including: the role that the student played, his/her own profession role, the role of other cooperative team members that other students played) in the IPE programme, students could immersively experience the interprofessional team working, and develop interprofessional knowledge and skills within the same simulation scenario. Here, the profession-role exchange was combined into an integrated learning method of cooperative learning and community service-learning, which we previously presented [7], in order to further improve the effectiveness of the designed extracurricular IPE activity. This study used the previously designed IPE learning method of cooperative learning and community service-learning as the control, and studied the effect of the addition of profession-role exchange as the intervention on the students’ attitudes towards interprofessional collaboration and their role understanding. The results showed a significant facilitating effect on the respondents’ orientation toward interprofessional team work and the role perceptions of team members in both the intervention group and the control group. Importantly, this effect is more pronounced after experiencing the addition of profession-role exchange than after experiencing with the control IPE activity without profession-role exchange component. In the control group, cooperative learning in which students were required to work together to learn and discuss in small-group learning situations was integrated prior to an interprofessional team-based community service. Cooperative learning experience helped students to prepare the knowledge and skills required in the subsequent community service, clarify their own professional roles, develop the team structure and a better understanding of the other professions’ roles, thus could encourage shared thinking among team members and lay the foundation for interprofessional community service learning [7]. In the intervention group, the addition of interprofessional role-exchange intervention offered a perspective taking experience that allowed students to understand other professionals’ roles in the practical team-based community health-care work, to reflect on their own past professional skills, as well as to understand the importance of interprofessional communication and collaboration. Moreover, this profession-role exchange experience provided opportunities for students to actually apply the knowledge and skills which they had prepared during the cooperative learning activities.

In this study, three survey instruments for attitudes toward interprofessional clinical collaboration, including SATP^2^C [21], JSAPNC [22] and a self-developed 25-item scale [23], were used to measure the students’ attitudes toward physician- pharmacist, physician-nurse and nurse-pharmacist collaborations, respectively. The profession-role exchange significantly enhance the scores in some extracted factors, especially “Responsibility and accountability” and “Shared authority” in SATP^2^C; “Nurse’s autonomy” and “Physician’s dominance” in JSAPNC; “Roles/responsibilities for collaborative practice” and “Nurses’ experience of pharmacist’s role in drug treatment” in the nurse-pharmacist collaboration survey instrument, which reflected the respondents’ professional role clarification. Moreover, the results from “Roles and responsibilities” sub-scale of RIPLS also indicated that the students’ understanding of roles of their own and other interrelated professions was further fostered after participating in the profession-role exchange experiences. These data showed that the experiences of students in role simulation as their cooperative team members in the teamwork situation were effective in strengthening the role identity and the communication among professions in the clinical setting. In their role as collaborators, health-care students have an opportunity to observe their own role in interprofessional collaboration from a new perspective, decide what elements to adopt in their own future clinical practice, and integrate their past professional knowledge and experiences to a novel situation for a creative solution.

Therefore, the combine of profession-role exchange with the cooperative learning-based community service IPE activity could further help students to develop the role perceptions of other health-care professionals, as well as hone their self-awareness, enhance interprofessional collaborative skill acquisition in a safe environment, thus prepare students for future real interprofessional contacts in a collaborative environment, allow for optimal contributions from all team members in providing holistic patient care. The integration of profession-role exchange component in an interprofessional student team-based community health service-learning experience could be used as a comprehensive extracurricular IPE tool for improving the role awareness of health-care students. This study provided a referential model for the professional role assignment during the further simulated IPE programs.

This study contained some limitations. SATP^2^C, JSAPNC and a previously self-developed 25-item scale were respectively chosen to detect students’ attitudes toward physician-pharmacist, physician-nurse and nurse-pharmacist collaboration. The “Roles and responsibilities” sub-scale of RIPLS was used to measure the overall role clarification. Although previous studies [21–24] have provided support for the validity and reliability of these measurement tools, the psychometric integrity of the Chinese and English bilingual versions of these instruments used in this study is still limited. Therefore, the psychometric properties of these chosen instruments as well as the internal consistency among items within each questionnaire measure should be further critically appraised. Because attitudes are only a proxy for behavior, it might be more ideal to demonstrate that this program can improve the communications and collaborative working in the workplace. However, there is still no quantitative method to measure the interprofessional collaboration behavior. Moreover, a total of 60 students were recruited in this study. In the studied university, there are about 300 medical students, 60 pharmacy students and 60 nursing students at each grade level. Therefore, the overall proportion of participants is less than 15%. And because the study participants are volunteers, it is unclear whether they represent all students. Furthermore, subjects were not randomly assigned to intervention or control group, so they were fully aware of the group they were in, which possibly caused socially-desired responses. In addition, the intervention of profession-role exchange was conducted only once. The small sample size and single-institution nature may limit the generalizability of the results. Further research and replication of this study with a larger sample of students from multiple institutions are needed to strengthen our findings.

## Conclusions

Our study concluded that a profession-role exchange component introduced in an interprofessional student team-based community health service-learning experience allowed the health-care students to experience the perspectives of their partners in the clinical team work under the community-based learning model. The profession-role exchange intervention resulted in the enhancement of students’ role awareness in interprofessional collaboration, thus could be used as an effective IPE tool.

## Data Availability

The datasets used and/or analysed during the current study are available from the corresponding author on reasonable request.

## Abbreviations

IPE: Interprofessional education
JSAPNC: Jefferson scale of attitudes towards physician-nurse collaboration
RIPLS: Readiness for interprofessional learning scale
SATP^2^C: Scale of attitudes toward physician-pharmacist collaboration
SD: Standard deviation
